# Lipoma of Paraglottic Space in a Child: A Case Report

**Published:** 2014-10

**Authors:** Sayed Hamidreza Abtahi, Afrooz Eshaghian, Farzaneh Abootalebian

**Affiliations:** 1 *Department of Otorhinolaryngology, Isfahan University of Medical Sciences, Isfahan, Iran. *

**Keywords:** Lipoma, Paraglottic space, Preepiglottic space.

## Abstract

**Introduction:**

Lipomas of the larynx are very rare benign lesions; macroscopically, they resemble retention cysts, so their diagnosis is usually made after surgery.

**Case Report:**

A rare case of pediatric paraglottic space Lipoma in an 11-year-old boy is explained. The mass was mobile, soft, without fluctuation or pulsation. CT scan revealed a 5.7 cm cervical fat density with regional lymphadenopathy. After lateral neck incision, a mass located deep in the carotid artery, which was attached to the larynx and which extended to the paraglottic space, was excised completely. Pathologic evaluation revealed Lipoma without any evidence of malignant cells present.

**Conclusion:**

This rare differential diagnosis for neck masses in pediatric population should be considered.

## Introduction

Benign mesenchymal neoplasms of the larynx include a wide variety of pathologies. One of the rare diagnoses is neoplasm of adipose origin, like Lipoma (0.6%) (1). Lipoma appears commonly in other parts of body, and is typically observed in patients who are more than 40 years old (2). It is more common in men (62.5 per cent). The most common site of head and neck Lipoma, is the posterior subcutaneous neck (3).

Lipoma originates from parts of the larynx in which fat tissue is normally located, like the aryepiglottic fold, the epiglottis, and false vocal folds (4).Though it has also been reported in true vocal cords. Lipoma is a slow growing mass, which is often present for a long time before diagnosis. It can be accompanied by respiratory symptoms and hoarseness. Treatment should be based on complete tumor removal, because it may grow again after incomplete excision. The size of tumor defines the surgical approach.(4) In this report, a rare case of pediatric paraglottic space Lipoma is explained.

## Case Report

The patient, who was presented to the clinic, was an 11-year-old boy with a neck mass he had acquired 10 years earlier. His family explained that this mass enlarged when drinking cold water and when exposed to cold weather. He had no previous major medical illnesses. 

**Fig 1(a,b,c:**
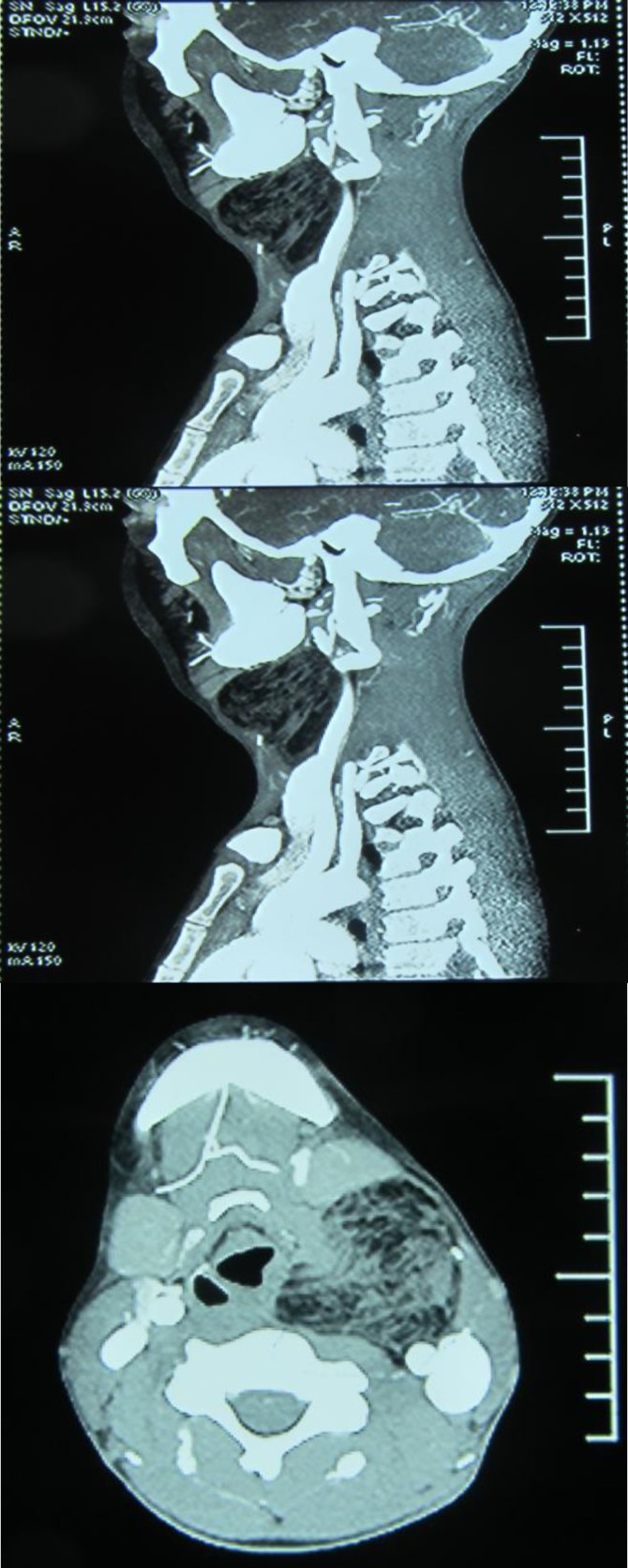
CT scan of neck with contrast (coronal, sagital, and axial view) revealed non-enhanced paraglottic space mass lesion

He had no inflammatory signs, dyspnea, swallowing disorder or hoarseness. The mass was mobile, soft, without fluctuation or pulsation. It was mobile during swallowing. Indirect laryngoscopy was normal. No other mass was observed during the neck examination. CT scan revealed a 5.7 cm cervical fat density with regional lymphadenopathy (Fig. 1(a,b,c)).

After lateral neck incision, a mass located deep in the carotid artery, which was attached to the larynx and which extended to the paraglottic space, was observed (Fig. 1). Because the mass was attached to supraglottic mucosa, it tore during tumor removal; then the epiglottic mucosa was repaired. The mass was excised completely, and a penrose drain was placed. The wound was repaired in 2 layers of subcutaneous and skin. Nasogastric tube placement was used for feeding after surgery, on the 4^th^ day after surgery. Pathologic evaluation revealed Lipoma without any evidence of malignant cells present.

## Discussion

In this case report, a rare case of pediatric paraglottic space Lipoma, that was uncommon regarding its location and the patient’s age, is explained.

These types of tumors reveal symptoms such as hoarseness, sensation of a foreign body in the throat, dyspnea, intermittent stridor, dysphagia, nocturnal cough, and dysphonia (5-9).

CT scan is the choice for preoperative imaging. A CT scan or MRI can correctly diagnose the Lipoma before surgery (3). 

There are some immunohistochemical markers such as S-100, vimentin, murine double minute 2 (MDM-2), and cyclin-dependent kinase 4 which can also be used for preoperative imaging (CDK4). (6)

The treatment recommended is based on complete tumor removal, because it may grow again after incomplete excision. The size of tumor defines the surgical approach (10). Tracheotomy may be necessary according to the patient and his/her tumor situation (11).

Long term follow-up of these patients is mandatory, because there is possibility of recurrence after a long period of time.(10) Also, it has been reported that tumors located in places that are difficult to approach, such as the retropharynx, were observed without surgical implementation in (12). 

## Conclusion

Lipomas of the larynx are very rare benign lesions. A rare case of pediatric paraglottic space Lipoma in an 11-year-old boy is explained. Such a rare differential diagnosis for neck masses in pediatric populations should be considered.
